# Supplementary material for: Evaluation of a Probability-Based Predictive Tool on Pathologist Agreement Using Urinary Bladder as a Pilot Tissue
Source: Vet Sci. 2022 Jul 18;9(7):367. doi: 10.3390/vetsci9070367 (PMC9323256; doi:10.3390/vetsci9070367)
Supplement: Supplementary file 1 [file vetsci-09-00367-s001.zip › vetsci-1801234-supplementary.pdf]

Supplementary Table S1: Diagnoses for every digital slide with the reference diagnosis, grouped by pathologist (P1-P4), under three different conditions (First, History and Tool).

Species	P1			P2			P3			P4			Reference diagnosis
	First	Hx	Tool	First	Hx	Tool	First	Hx	Tool	First	Hx	Tool	
Canine	Neo	Neo	Neo	Neo	Neo	Neo	Neo	Neo	Neo	Neo	Neo	Neo	Neoplasia - UC
Feline	Neo	Neo	Neo	Neo	Neo	Neo	Neo	Neo	Neo	.	.	.	Neoplasia - UC
Canine	Neo	Neo	Neo	Cys	Cys	Cys	Uro	Uro	Uro	.	.	.	Neoplasia - UC
Feline	Neo	Neo	Neo	Neo	Neo	Neo	Neo	Neo	Neo	Neo	Neo	Neo	Neoplasia - UC
Canine	Cys	Cys	Cys	Cys	Cys	Cys	Cys	Cys	Cys	.	.	.	Cystitis, mild
Feline	Cys	Cys	Cys	Cys	Cys	Cys	Cys	Cys	Cys	Cys	Cys	Cys	Cystitis secondary to lower motor neuron bladder
Canine	Cys	Cys	Cys	Cys	Cys	Cys	Cys	Uro	Uro	Uro	Uro	Uro	Urolithiasis - Cystitis + uroliths at necropsy
Canine	Cys	Cys	Oth[b]	Oth	Oth	Oth	Cys	Cys	Cys[d]	Neo	Neo	Neo	Neoplasia - Leiomyosarcoma
Canine	Uro	Uro	Uro	Cys	Cys	Cys	Cys	Uro	Uro	Uro	Uro	Uro	Urolithiasis - Cystitis + stones removed surgically
Feline	Uro	Uro	Uro[a]	Cys	Cys	Cys	Cys	Uro	Uro	Oth	Oth	Oth[l]	Cystitis, suspect FIC - urethral obstruction, no uroliths at necropsy
Feline	Uro	Uro	Uro[a]	Cys	Cys	Cys	Cys	Cys	Cys	Oth	Oth	Oth[l]	Cystitis, suspect FIC - clinical signs of stranguria. No stones or obstruction at necropsy
Feline	Uro	Uro[a]	Cys	Cys	Cys	Cys	Cys	Cys	Cys	Cys	Cys	Cys	Cystitis, suspect FIC - clinical urethral obstruction, no uroliths at necropsy
Canine	Cys	Cys	Cys	Cys	Cys	Cys	Cys	Cys	Cys	Cys	Cys	Cys	Cystitis
Canine	Norm	Norm	Norm	Norm	Norm	Norm	Cys	Cys	Cys	.	.	.	Normal
Feline	Norm	Norm	Norm	Oth	Oth	Oth[f]	Cys	Cys	Cys[d]	.	.	.	Neoplasia - Epitheliotropic lymphoma (metastasis)

Canine	Norm	Norm	Norm	Norm	Norm	Norm	Cys	Cys	Cys[d]	Norm	Norm	Norm	Normal
Canine	Norm	Norm	Norm	Oth	Oth	Oth[g]	Oth	Oth	Oth[k]	Oth	Norm	Norm	Normal
Canine	Cys	Norm	Cys[c]	Oth	Oth	Oth[g]	Cys	Cys	Cys	.	.	.	Normal
Feline	Uro	Uro	Uro[a]	Neo	Neo	Neo	Cys	Uro	Uro	Cys	Uro	Uro	Urolithiasis - Urethral obstruction and bladder rupture, uroliths at necropsy.
Feline	Cys	Cys	Cys[d]	Cys	Cys	Cys	Norm	Norm	Norm	Cys	Norm	Norm	Normal
Feline	Norm	Norm	Norm	Oth	Oth	Oth[h]	Norm	Norm	Norm	Uro	Norm	Norm	Normal
Feline	Uro	Uro	Oth[e]	Cys	Cys	Cys[i]	Cys	Cys	Cys[d]	Uro	Uro	Uro	Urolithiasis (cystotomy) + UTI (positive culture)
Canine	Uro	Uro	Uro	Cys	Cys	Cys[i]	Oth[j]	Uro	Uro	Uro	Uro	Uro	Urolithiasis
Feline	Uro	Uro	Uro	Cys	Cys	Cys	Cys	Uro	Uro	Uro	.	.	Cystitis, suspect FIC - clinical urethral obstruction, no uroliths at necropsy
Canine	Uro	Uro	Uro	Cys	Cys	Cys	Cys	Uro	Uro	Cys	.	.	Urolithiasis + UTI

Key: First = First read of the slide, with no animal information; Hx = Second read of the slide, with animal signalment and clinical history; Tool = Third read of the slide, with the predictive tool.

Cys = Cystitis; FIC = Feline idiopathic cystitis; Neo = Neoplasia; Norm = Normal; Oth = Other diagnosis; UC = Urothelial carcinoma; Uro = Urolithiasis. Full stop indicates slides that were unable to be assessed due to technical issues.

Comments from the pathologists: [a] Suspected FIC cases, called these urolithiasis as many have plugs that act similarly to stones; [b] Polypoid cystitis, mild inflammation, secondary to stones; [c] Follicular cystitis; [d] Normal/minimal clinically insignificant cystitis; [e] Marked hemorrhage; [f] Urothelial hyperplasia with glandular metaplasia; [g] Urothelial hyperplasia; [h] Focal mucosal ulceration, intraluminal hemorrhage; [i] Obstruction/uroliths?; [j] Poorly scanned, had trouble evaluating; [k] Myositis; [l] Mucosal hemorrhage and necrosis.

Supplementary Table S2: The sensitivity and specificity of the diagnosis by the four pathologists under the various slide reading conditions, and digital versus glass slides.

	Reference Diagnosis			
	Cystitis	Neoplasia	Normal	Urolithiasis
		**All data**		
Sensitivity	0.696	0.648	0.564	0.500
Specificity	0.695	0.976	0.978	0.801
		**No animal information**		
Sensitivity	0.696	0.583	0.511	0.383
Specificity	0.654	0.98	0.978	0.763
		**Signalment and history**		
Sensitivity	0.643	0.639	0.596	0.562
Specificity	0.718	0.980	0.971	0.791
		**Predictive tool probabilities**		
Sensitivity	0.750	0.727	0.587	0.553
Specificity	0.714	0.966	0.985	0.852
		**Glass**		
Sensitivity	0.774	0.778	0.597	0.417
Specificity	0.650	0.974	0.995	0.854
		**Digital**		
Sensitivity	0.619	0.580	0.529	0.586
Specificity	0.734	0.977	0.964	0.756

The sensitivity and specificity of the diagnostic approaches used by the four test pathologists is presented in **Error! Reference source not found.**. Overall, a diagnosis of cystitis had the highest sensitivity (69.6 %) but lowest specificity (69.5 %) of all the diagnoses. The sensitivity of diagnosing urolithiasis was unsurprisingly highly influenced by the additional of clinical history. When comparing glass slides to digital slide images, cystitis diagnosis had higher sensitivity and lower specificity with glass slides; neoplasia had a higher sensitivity and comparable specificity; normal had higher sensitivity and specificity with glass slides; and the urolithiasis diagnosis had lower sensitivity but higher specificity when using glass slides compared to digital slide images.

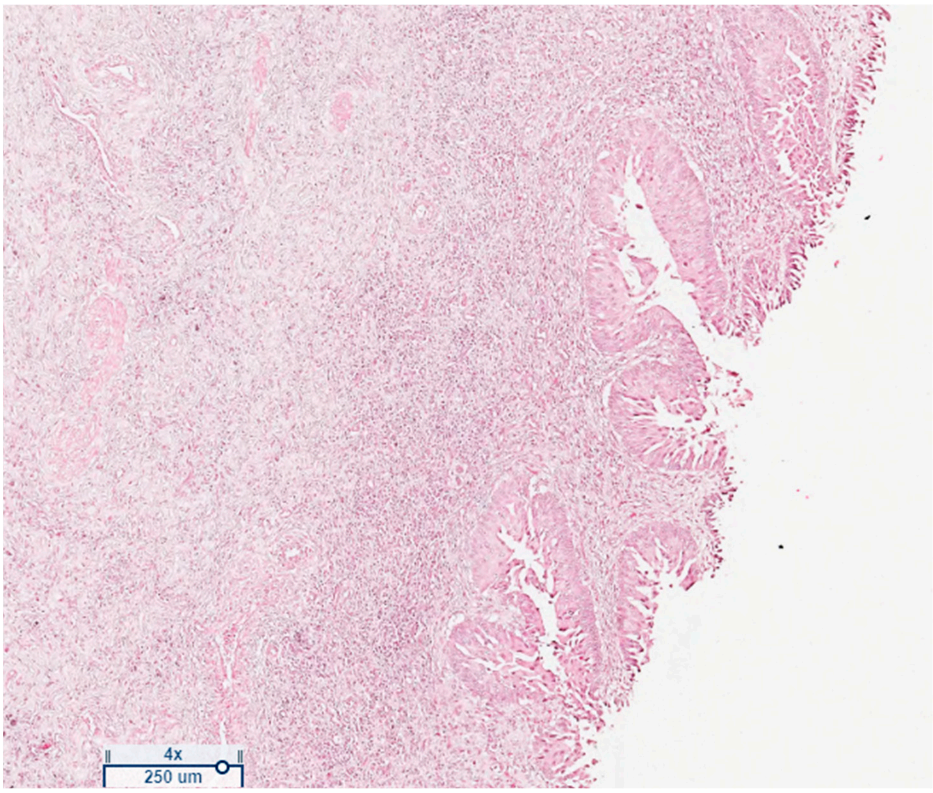

Supplementary Figure S1: A case of cystitis in a cat from the whole slide image case library (40x magnification).

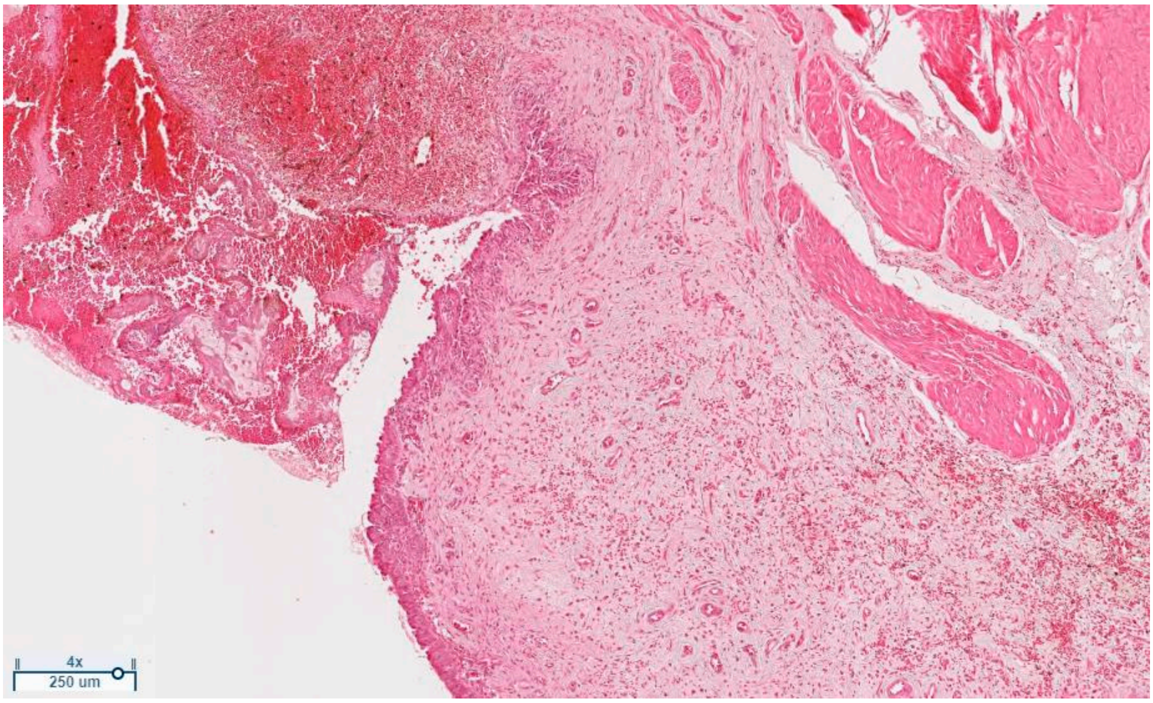

Supplementary Figure S2: A case of urolithiasis in a dog from the whole slide image case library. This slide exhibits the typical histological features of urolithiasis – urothelial ulceration and submucosal haemorrhage and oedema (40x magnification).

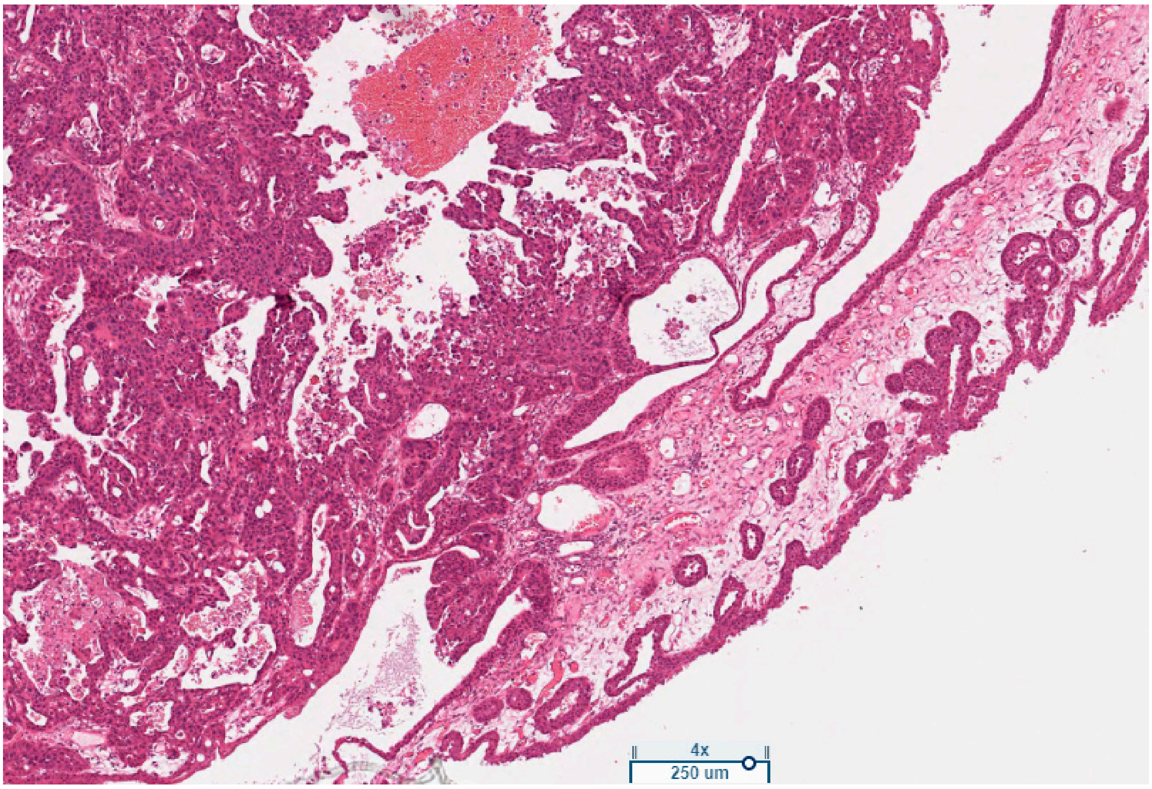

Supplementary Figure S3: A case of bladder neoplasa (urothelial carcinoma) in a cat from the whole slide image case library (40x magnification).

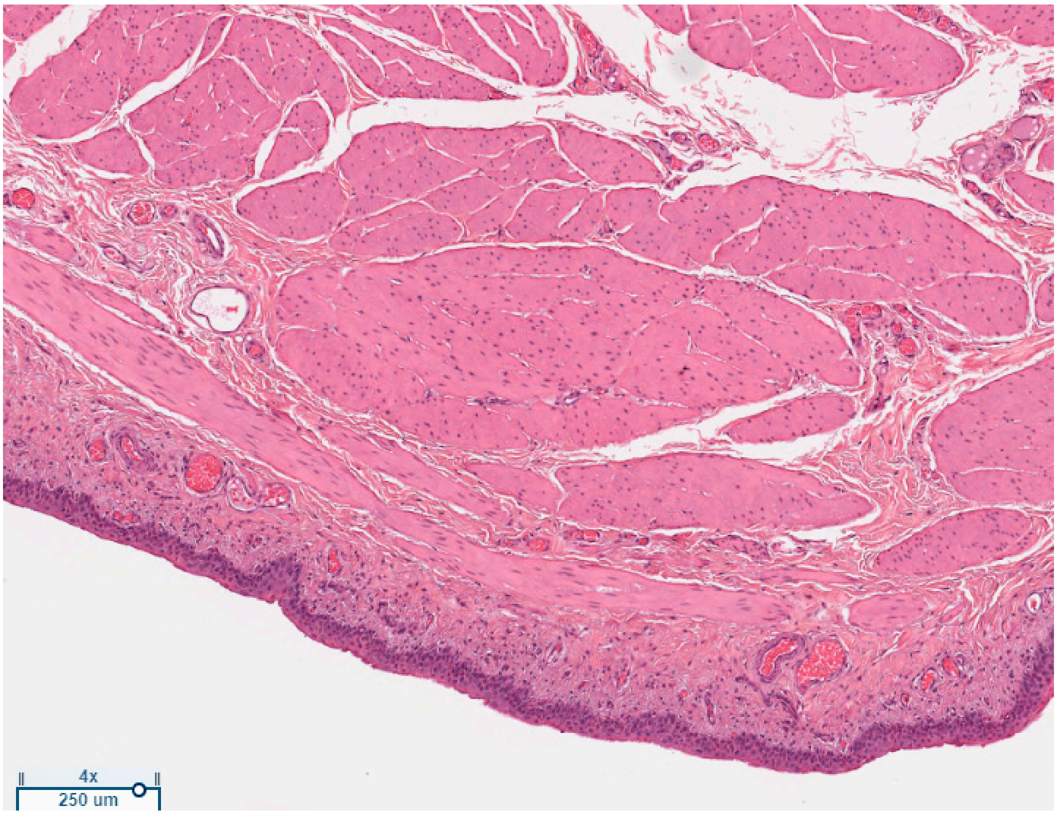

Supplementary Figure S4: A normal canine bladder from the whole slide image case library (40x magnification).
